# Molecular Basis for the Differential Function of HAVCR1 Mucin Variants

**DOI:** 10.3390/biomedicines12112643

**Published:** 2024-11-19

**Authors:** Abdolrahim Abbasi, Maria Isabel Costafreda, Angela Ballesteros, Jerome Jacques, Cecilia Tami, Mohanraj Manangeeswaran, José M. Casasnovas, Gerardo Kaplan

**Affiliations:** 1 Center for Biologics Evaluation and Research, U.S. Food and Drug Administration, Silver Spring, MD 20993, USA; abbasi_71@yahoo.com (A.A.); angela.ballesteros@nih.gov (A.B.); jerome.jacques@usp.org (J.J.); tamim@gene.com (C.T.); mohanraj.manangeeswaran@fda.hhs.gov (M.M.); 2 Department of Macromolecular Structures, Centro Nacional de Biotecnología and Consejo Superior de Investigaciones Científicas (CNB-CSIC), Campus Cantoblanco, 28049 Madrid, Spain; jcasasnovas@cnb.csic.es

**Keywords:** CD365, TIM1, phosphatidylserine receptor, mucin polymorphisms, binding of apoptotic cells, signal transduction, PI3K, AKT, mTOR

## Abstract

**Background/Objectives**: The hepatitis A virus (HAV) cellular receptor 1 (HAVCR1) is a type I integral membrane glycoprotein discovered in monkeys and humans as a HAV receptor. HAVCR1 contains an N-terminal immunoglobulin-like variable domain (IgV) followed by a mucin-like domain (Muc), a transmembrane domain, and a cytoplasmic tail with a canonical tyrosine kinase phosphorylation site. The IgV binds phosphatidylserine on apoptotic cells, extracellular vesicles, and enveloped viruses. Insertions/deletions at position 156 (156ins/del) of the Muc were associated in humans with susceptibility to atopic, autoimmune, and infectious diseases. However, the molecular basis for the differential function of the HAVCR1 variants is not understood. **Methods**: We used mutagenesis, apoptotic cell binding, and signal transduction analyses to study the role of the 156ins/del in the function of HAVCR1. **Results**: We found that the HAVCR1 variant without insertions at position 156 (156delPMTTTV, or short-HAVCR1) bound more apoptotic cells than that containing a six amino acid insertion (156insPMTTTV, or long-HAVCR1). Furthermore, short-HAVCR1 induced stronger cell signaling and phagocytosis than long-HAVCR1. **Conclusions**: Our data indicated that the 156ins/del determine how the IgV is presented at the cell surface and modulate HAVCR1 binding, signaling, and phagocytosis, suggesting that variant-specific targeting could be used as therapeutic interventions to treat immune and infectious diseases.

## 1. Introduction

**Figure 1:**
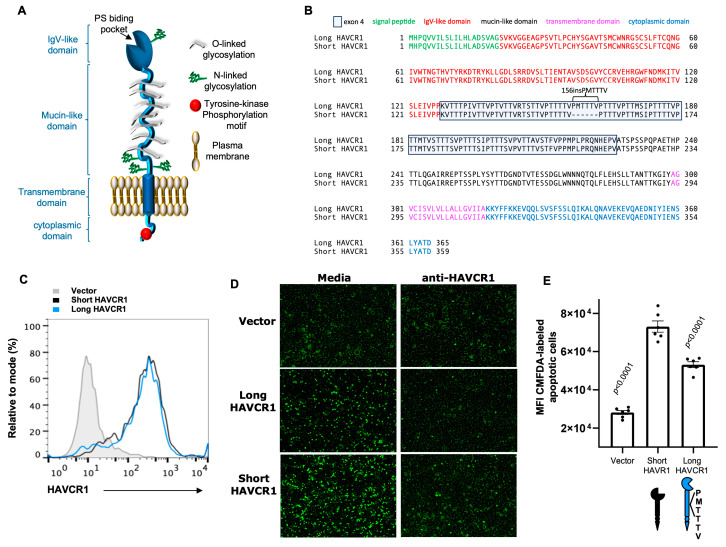
Insertion/deletion variants in the mucin-like (Muc) domain of HAVCR1 modulate binding of apoptotic cells to the immunoglobulin-like (IgV) domain. (**A**) Schematic representation of HAVCR1. HAVCR1 is a class I integral membrane glycoprotein with an IgV N-terminal domain containing a MILIBS (PS-binding pocket), which is extended from the cell surface by a Muc domain anchored to the plasma membrane by a transmembrane domain and a cytoplasmic tail. (**B**) Amino acid sequence alignment of short-HAVCR1 (156delMPTTTV) and long-HAVCR1 (156insPMTTTV) variants. Amino acids (one letter code) aligned using the ClustalW program. Signal peptide (green letters), IgV domain (red letters), Muc domain (black letters) with exon 4 residues highlighted in a grey box and deleted amino acids shown as dashes, transmembrane domain (magenta letters), and cytoplasmic domain (blue letters). (**C**) Flow cytometry analysis of the expression of HAVCR1 at the cell surface of 293 H cells stably transfected with the cDNA of short-HAVCR1 (black line), long-HAVCR1 (blue line), or vector (filled grey histogram) stained with anti-HAVCR1 mAb 1D12. (**D**) Binding of apoptotic cells to 293 H stable transfectants expressing HAVCR1. Subconfluent monolayers of stable 293 H cell transfectants expressing long-HAVCR1, short-HAVCR1, or vector were treated with 1 μg/mL anti-HAVCR1 mAb 1D12 or media at room temperature for 30 min. CMFDA-labeled apoptotic Jurkat cells were added to the monolayers, incubated for 30 min at RT, washed extensively, and visualized in an Axiovert 200 fluorescence microscope. Micrographs showing CMFDA fluorescence (green) were taken at 200× using AxioVision software version 4.8. (**E**) Quantitation of apoptotic cell binding to 293 H cell transfectants. Binding of CMFDA-labeled apoptotic cells to monolayers treated with media as in (**D**) was analyzed in a fluorescence plate reader. Data are mean ± s.e.m., *n* = 6 from three independent experiments. *p*-values between short-HAVCR1 and the other cell transfectants were determined by one-way ANOVA with Dunnett’s test analysis.

The hepatitis A virus (HAV) receptor 1 (HAVCR1) is a type I integral-membrane glycoprotein composed of an N-terminal immunoglobulin variable-like domain (IgV) extended from the cell surface by a mucin-like domain (Muc), a transmembrane domain, and a cytoplasmic tail with a canonical tyrosine kinase phosphorylation site (Figure 1A). HAVCR1 chemical activation or crosslinking with monoclonal antibodies induce phosphorylation of the cytoplasmic tail of HAVCR1 and co-stimulation of T-cell receptor (TCR) [1,2]. Activation of HAVCR1 induces the phosphoinositide 3-kinase/protein kinase B (PI3K/Akt) pathway, which is a conserved signaling network interconnected with other signaling pathways that modulates cell survival, growth, and differentiation (for a review, see [3]). 

HAVCR1, the first described member of a conserved family of receptors in vertebrates, was initially identified as a HAV receptor in African green monkey kidney cells [4] and subsequently in human cells [5]. In humans, *HAVCR1* and its homologs *HAVCR2* and *TIMD4* are located in chromosome 5q33.2 and function as phosphatidylserine (PS) receptors [6,7,8,9]. PS exposed on the plasma membrane outer leaflet of apoptotic cells binds to a conserved pocket in the HAVCR IgV, termed the metal-ion-dependent ligand binding site (MILIBS) [8], resulting in signaling events [10] and phagocytosis of apoptotic cells [7,9,11]. HAVCR1 was rebranded as kidney injury 1 (KIM1) because of its role in kidney disease [12,13] and T-cell immunoglobulin mucin 1 (TIM1) because of its role as a co-stimulatory molecules of T cells [14,15].

HAVCR1 serves as a functional HAV receptor for naked particles and infectious exosomes produced by HAV-infected cells [4,5,16,17,18,19]. Through the interaction with PS on viral envelopes [20], HAVCR1 also enhances the infectivity of several enveloped viruses functioning as a cell entry factor [21,22,23,24,25]. However, in HIV-infected cells, HAVCR1 has the opposite effect blocking the release of viral particles and reducing infection of naive cells [26]. Extracellular vesicles, which mediate intercellular communications [27] and often contain PS at their surface, can also use HAVCR1 for binding and cargo delivery [19]. 

HAVCR1 is expressed in several cell types including hepatocytes, tubular epithelial kidney cells [12], mast cells [28], and lymphoid cells [15], playing an important role in the regulation of allergy, autoimmunity, infectious diseases, graft versus host disease, and cancer [15,29,30]. Interestingly, HAVCR1 is constitutively expressed in human natural killer T (NKT) [31], CD4+ T-regulatory (Treg) [10], and B-regulatory (B-reg) [32] cells, and contributes to the modulation of immune responses. 

*HAVCR1* is a highly polymorphic gene [33,34]. Single nucleotide polymorphisms (SNPs) and insertion/deletion (ins/del) variants in the *HAVCR1* promoter, IgV, and Muc have been associated in some but not all studies with the development of allergic [35,36,37,38,39,40,41,42,43,44,45], autoimmune [46,47,48,49,50,51], cardiac [52], and infectious [31,42,53,54,55,56,57,58] diseases. As expected, *HAVCR1* polymorphisms in the promoter region affected transcription [48,49] and in the IgV-like binding domain controlled the interaction with ligands [59]. However, it is not understood how Muc polymorphisms affect the function of HAVCR1. 

Exon 4 of *HAVCR1* encodes the Muc region that contains 13 consecutive repeats of six amino acids of the consensus sequence PTTTTL [5]. The insertion of an extra hexamer repeat in the HAVCR1 Muc, initially described by McIntire and colleagues [60], is associated with atopy protection. These authors described the polymorphism as an insertion of amino acids MTTTVP at position 157 of HAVCR1 (157insMTTTVP). This insertion is within the repeat portion of the HAVCR1 Muc flanked by proline residues, which could also be described as the insertion of amino acids PMTTTV at position 156 of HAVCR1 (156insPMTTTV) by placing the P residue at the beginning of the insertion. Herein, we will refer to this polymorphism as the 156insPMTTTV insertion to follow the nomenclature established in the two original papers describing HAVCR1 [4,5], which was used to guide the mutagenesis analysis in this work. The HAVCR1 156insPMTTTV polymorphism results in a longer receptor (long-HAVCR1) compared to the variant lacking the insertion (short-HAVCR1). 

In the present study, we analyzed the effect of exon 4 polymorphisms in the HAVCR1 natural function of binding apoptotic cells and inducing cell signaling. Our study focused on human HAVCR1 and its direct activation by apoptotic cells and not on the co-stimulatory function in B- and T-cells. The mouse ortholog of HAVCR1 (havcr1) also has short and long variants, but the mucin region of these orthologs is not conserved and lacks hexamer repeats [14,15]. Therefore, havcr1 was not analyzed in the present study due to the significant structural differences with the HAVCR1 Muc. We observed that binding and phagocytosis of apoptotic did not require phosphorylation of the HAVCR1 cytoplasmic tail. Importantly, we showed that cells expressing short-HAVCR1 bind more apoptotic cell and induce a stronger cell signaling than cells expressing long-HAVCR1. Our data indicated that the 156ins/del polymorphisms in the HAVCR1 Muc modulate how the IgV binding domain is presented at the cell surface. Because short-HAVCR1 is associated with rheumatoid arthritis (RA) [49], a Th1- and Th17-mediated disease, and long-HAVCR1 is associated with atopy [60,61], a Th2-mediated disease, our data suggest that the strong cell signaling mediated by short-HAVCR1 skews the immunity towards Th1 and Th17 responses, whereas the weaker long-HAVCR1 signaling favors Th2 responses. 

## 2. Materials and Methods

### 2.1. Cells, Antibodies, Reagents, Liposomes, and Viruses

Human embryonic kidney 293 H cells (ThermoFisher Scientific, Waltham, MA, USA) were maintained in Dulbecco’s modified Eagle medium (DMEM, ThermoFisher Scientific, Waltham, MA, USA) supplemented with 10% fetal bovine serum (FBS, Cytiva, Marlborough, MA, USA), 2 mM L-glutamine (ThermoFisher Scientific, Waltham, MA, USA), and 1% penicillin-streptomycin (ThermoFisher Scientific, Waltham, MA, USA). T-cell leukemia Jurkat E6-1 cells (TIB-152, ATCC, Manassas, VA, USA) were maintained in RPMI medium supplemented with 10% FBS, 2 mM L-glutamine, and 1% penicillin-streptomycin.

Mouse anti-human HAVCR1 mAb 1D12 (Biolegend, Inc., San Diego, CA, USA) directed against the IgV domain of HAVCR1 was used to block binding of apoptotic cells and as a primary antibody in flow cytometry analysis. PE-labeled anti-mouse polyclonal IgG (Southern Biotechnology, Birmingham, AL, USA) was used as secondary antibody. 

Etoposide (MilliporeSigma, Burlington, MA, USA) was used to induce apoptosis in Jurkat cells. Cell Tracker Green 5-chloromethylfluorescein diacetate (CMFDA) (Thermo Fisher Scientific, Waltham, MA, USA), and pH-sensitive pHrodo Red (Thermo Fisher Scientific, Waltham, MA, USA) fluorescent dyes were used to stain apoptotic Jurkat cells. 

Liposomes were prepared by the lipid extrusion method with an Avanti Mini-Extruder kit and a 30 nm membrane (AvantiPolar Lipids, Birmingham, AL, USA) as recommended by the manufacturer. Phosphatidylcholine (PC) and phosphatidyserine (PS) were purchased from AvantiPolar Lipids, Birmingham, AL, USA. PC liposomes were prepared using 100% PC, whereas PS:PC liposomes were prepared at a molar ratio of 7:3. 

### 2.2. Squences, Plasmids, and Mutagenesis

pEF6/V5-His A (ThermoFisher Scientific, Waltham, MA, USA), a eukaryotic expression plasmid containing a blasticidin resistance gene, was used as vector control for experiments. The cDNA of short-HAVCR1 without insertions at position 156 of the MUC (HAVCR1 156delPMTTTV, GenBank AF043724) or long-HAVCR1 containing the 6 amino acid insertion PMTTTV at position 156 of the MUC (HAVCR1 156insPMTTTV, National Center for Biotechnology Information. ClinVar; VCV000004270.1) were cloned into the polylinker of pEF6/V5-His A. The resulting plasmids containing the short- or long-HAVCR1 cDNA under the control of the human EF1 alpha promoter were termed pEFshort-HAVCR1 and pEFlong-HAVCR1, respectively.

HAVCR1 insertions, deletions, and single amino acid changes were generated by PCR mutagenesis. To do so, HAVCR1 cDNA fragments were replaced with PCR fragments generated with synthetic oligonucleotides containing the desired mutations. For insertions in the mucin region of HAVCR1, mutated PCR fragments were cloned into pEFshort-HAVCR1. For Y to A mutations and deletions in the cytoplasmic tail of HAVCR1, mutated PCR fragments were cloned into pEFlong-HAVCR1. Nucleotide sequences from all constructs were verified by automated Sanger sequencing analysis.

### 2.3. Transfection of Cells

The 293 H cells were transfected using Fugene 6 Transfection Reagent (Promega Corp, Madison, WI, USA) as facilitator following the manufacturer’s recommendations. Jurkat E6-1 cells were electroporated using the Amaxa Nucleofector^®^ system and the Cell Line Nucleofector^®^ Kit V (Lonza, Inc., Allendale, NJ, USA) as suggested by the manufacturer. Stable cell transfectants were selected with 5 µg/mL of blasticidin (ThermoFisher Scientific, Waltham, MA, USA) and single-cell clones were obtained by limiting dilution in 96-well plates. Cell surface expression of HAVCR1 in cells stained with anti-HAVCR1 mAb 1D12 and secondary PE-labeled anti-mouse IgG was analyzed by flow cytometry in a Guava EasyCyte instrument (MilliporeSigma, Burlington, MA, USA) using FlowJo software version 10 (Becton, Dikinson and Co., Ashland, OR, USA). Cell clones that expressed similar levels of HAVCR1 at the cell surface were selected to perform further studies.

### 2.4. Apoptotic Cell Binding Assays

Binding of apoptotic cells to cell monolayers was performed as described [10]. Briefly, 293 H cells transfected with HAVCR1 variants or vector were seeded in collagen-coated 12-well plates 24 h prior to the experiment. Jurkat cells were treated with 50 µM of etoposide during 8 h at 37 °C to induce apoptosis, washed, stained with 5 µmol/L of CMFDA for 15 min at 37 °C in serum free media, and washed extensively to remove excess dye. Subconfluent monolayers of 293 H transfectants were incubated with 10^6^ CMFDA-labeled apoptotic Jurkat cells per well at RT for 30 min and washed thrice. CMFDA fluorescence from bound apoptotic cells was quantified in a Sinergy HT fluorescence plate reader (BIOTEK Corp., Winooski, VT, USA) using 485/20 excitation and 528/20 emission filters. Binding of apoptotic cells was also visualized under an inverted Zeiss Axiovert 200 fluorescence microscope (Carl Zeiss Microscopy, LLC, Thornwood, NY, USA) at 200× using a fluorescein isothiocyanate filter, and micrographs were taken using AxioVision software (Carl Zeiss Microscopy, LLC, Thornwood, NY, USA).

For the combined binding and endocytosis assay, apoptotic Jurkat cells were stained with 5 µmol/L of CMFDA to track cell surface binding and 0.5 µg/mL of pH-sensitive pHrodo Red to track phagocytosis of the bound apoptotic cells. Subconfluent monolayers of 293 H cell transfectants in collagen-coated 12-well plates were incubated with 10^6^ apoptotic CMFDA/pHrodo Red-labeled Jurkat cells per well for 30 min at RT, washed thrice, and incubated for 1 h at 37 °C. CMFDA and pHrodo Red fluorescence were quantified after 30 min incubation at RT and 1 h incubation at 37 °C, respectively. Plates were read in a Sinergy HT fluorescence plate reader using 485/20 excitation and 528/20 emission filters for CMFDA, and 530/25 excitation and 590/35 emission filters for pHrodo Red.

### 2.5. Multiplex Cell Signaling Assay

Phosphorylation of PI3K, AKT, and the mammalian target of rapamycin (mTOR) was assessed using a magnetic bead multiplex assays based on Luminex technology. The Bio-Plex Pro™ Cell Signaling Reagent Kit (Bio-Rad Laboratories, Hercules, CA, USA) with analyte-specific magnetic beads and detection antibody sets for PI3Kp85 (Tyr^458^), AKT(Ser^473^), and mTOR(Ser^2448^) were used to detect phosphorylated proteins. Housekeeping protein assays for GAPDH or G6PI (Bio-Rad Laboratories, Hercules, CA, USA) were used to normalize the results.

For the activation of 293 H cell transfectants with liposomes or apoptotic cells, sub-confluent monolayers of 293 H cell transfectants expressing short-HAVCR1, long-HAVCR1, or vector in collagen-coated 12-well plates were treated with 100 μM of PS:PC or PC liposomes or with 2–4 × 10^5^ apoptotic cells per well. For the activation of Jurkat cells, transfectants expressing short-HAVCR1, long-HAVCR1, or vector were grown at densities below 5 × 10^5^ per ml to prevent self-activation. Jurkat cell transfectants (10^6^ cells) were treated with 100 μM of PS:PC or PC liposomes or 2–4 × 10^5^ apoptotic cells. Liposome and apoptotic cell treatments were performed at 37 °C under 5% CO_2_ for 0 min or 30 min and washed with cold PBS. Cell signaling assays were performed as recommended by the manufacturer. Briefly, cell extracts were prepared by treatment with lysis buffer supplemented with protease/phosphatase inhibitors for 20 min at 4 °C, cell lysates were centrifuged at 12,000 rpm for 10 min, and pellets were discarded. Analyte-specific magnetic bead sets for PI3Kp85, Akt, and mTOR (10–20 μL each) were combined, washed twice with washing buffer, incubated overnight with 50 µL of cell lysate at room temperature, and washed 3 times in an automated wash station (Bio-Rad Laboratories, Hercules, CA, USA). After washing, magnetic beads were incubated with 25 µL of combined detection antibodies for 30 min at room temperature, washed, incubated with 50 µL of streptavidin-PE (SAPE) for 10 min, washed, and resuspended in assay buffer. The magnetic beads were read on a Bio-plex 200 system (Bio-Rad Laboratories, Hercules, CA, USA) and analyzed with Bio-plex manager 6.1.1 software. Phosphoprotein levels in 293 H and Jurkat cell extracts were normalized with GAPDH and G6PI protein levels, respectively.

### 2.6. Amino Acid Sequence Alignments

Amino acid sequences of HAVCR1 alleles and mutants were aligned using the Muscle program in MacVector version 18.2.5 software package (MacVector, Inc., Apex, NC, USA). 

### 2.7. Statistical Analyses

All experiments were conducted at least three times using 2–3 technical replicates. Statistical analysis was performed using Prism version 9 software (GraphPad Software, Boston, MA, USA).

Binding of CMFDA-labeled apoptotic cells to 293 H cell transfectants was analyzed by one-way ANOVA with Dunnett’s test for multiple comparisons.

Signal transduction of 293 H and Jurkat cells activated with liposomes or apoptotic cells was analyzed by two-way ANOVA with Tukey’s test for multiple comparisons. 

Regression analysis of apoptotic cell binding and endocytosis was performed using the Excel program (Microsoft Corp., Redmond, WA, USA).

## 3. Results

### 3.1. HAVCR1 Polymorphisms in the Mucin-Like Domain (Muc) Modulate Binding of Apoptotic Cells to the IgV Binding Domain

PS binds to the MILIBS (PS binding pocket) of the HAVCR1 IgV [8] (Figure 1A). Apoptotic cells display PS on the plasma membrane outer leaflet as a damage-associated distress pattern (DAMP) that functions as an “eat me” signal recognized by pattern recognition receptors (PRR), such as HAVCR1, triggering phagocytosis of the apoptotic cells (for reviews, see [62,63]). To determine whether natural variants of HAVCR1 (Figure 1B) differentially bind apoptotic cells, we compared binding of CMFDA-labeled apoptotic Jurkat cells to 293 H stable transfectants expressing similar levels of short-HAVCR1 or long-HAVCR1 at the cell surface (Figure 1C). Fluorescence microscopy analysis revealed that 293 H cells expressing short-HAVCR1 bound more CMFDA-labeled Jurkat cells than cells expressing long-HAVCR1, whereas vector transfectants bound background levels of apoptotic cells (Figure 1D, left panels). Treatment with anti-HAVCR1 mAb 1D12, which binds to the IgV domain of HAVCR1 and blocks the interaction of PS with the IgV, prevented binding of CMFDA-labeled apoptotic cells to short- and long-HAVCR1 transfectants but had no effect on vector transfectants (Figure 1D, right panels). These binding data indicated that apoptotic cells bound specifically to HAVCR1 expressed at the cell surface of short- and long-HAVCR1 cell transfectants. Quantitation of apoptotic cell binding to the cell surface of the 293 H cell transfectants showed that short-HAVCR1 bound significantly more apoptotic cells than long-HAVCR1 as assessed by an apoptotic-cell binding fluorescence assays in 12-well plates (Figure 1E). Taking together, these results indicate that polymorphisms in the HAVCR1 Muc modulates the function of the IgV binding domain, and suggest that the 156insPMTTTV polymorphism affected the conformation of HAVCR1 at the cell surface limiting the availability of the PS-binding pocket to apoptotic cells.

### 3.2. Moving the PMTTTV Insertion from Position 156 to 150 or 162 Also Reduce Binding of Apoptotic Cells to the HAVCR1 IgV

**Figure 2:**
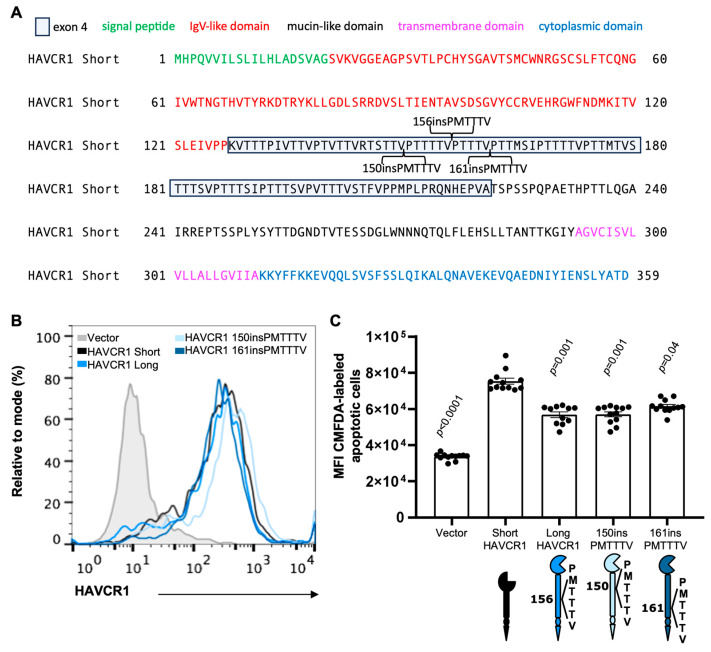
Positional insertion of PMTTTV to adjacent Muc repeats also reduced binding of apoptotic cells. (**A**) Amino acid sequence of short-HAVCR1 showing the PMTTTV insertion at position 156 (156insPMTTTV, long-HAVCR1 variant), 150 (150insPMTTTV), and 162 (162insPMTTTV). (**B**) Flow cytometry analysis of the expression of HAVCR1 at the cell surface of 293 H cells stably transfected with the cDNA of short-HAVCR1 (black line), long-HAVCR1 (blue line), HAVCR1 150insPMTTTV (light blue line), HAVCR1 161insPMTTTV (dark blue line), or vector (filled grey histogram) stained with anti-HAVCR1 1D12 mAb. (**C**) Binding of apoptotic cells to 293 H cells expressing HAVCR1. Subconfluent monolayers of stably transfectants from (**B**) were treated with CMFDA-labeled Jurkat apoptotic cells for 30 min at RT. Monolayers were extensively washed and fluorescence was measured in a microplate reader. Data are mean ± s.e.m., *n* = 12 biological replicates from four independent experiments with three biological replicates each. *p*-values between long-HAVCR1 and the other cell transfectants were determined by one-way ANOVA with Dunnett’s test analysis.

The insertion of multiple amino acid residues in natural occurring variants of HAVCR1 is restricted to position 156 of the Muc repeat region [34]. To analyze whether there is a positional restriction for the PMTTTV insertion to reduce binding of apoptotic cells to the IgV, we inserted PMTTTV in adjacent repeats at positions 150 (150insPMTTTV) or 161 (161insPMTTTV) of the Muc (Figure 2A). Stable 293 H cell transfectants expressing similar levels of short-, long-, 150insPMTTTV-, or 161insPMTTTV-HAVCR1 were selected by flow cytometry (Figure 2B). The 293 H transfectants expressing HAVCR1 150insPMTTTV, 156insPMTTTV (long-HAVCR1), and 161insPMTTTV bound similar levels of apoptotic cells (Figure 2C). As expected, short-HAVCR1 bound more apoptotic cells than any of the constructs containing the PMTTTV insertion at different positions. These binding data indicate that the PMTTTV insertion has no positional requirement within the 150 to 161 segment to reduce binding of apoptotic cells to the IgV, and suggest that the restriction to position 156 in natural variants is due to other unknown determinants.

### 3.3. The Insertion of a P Residue at Position 156 of Short-HAVCR1 Is Sufficient to Reduce Binding of Apoptotic Cells to the IgV

**Figure 3:**
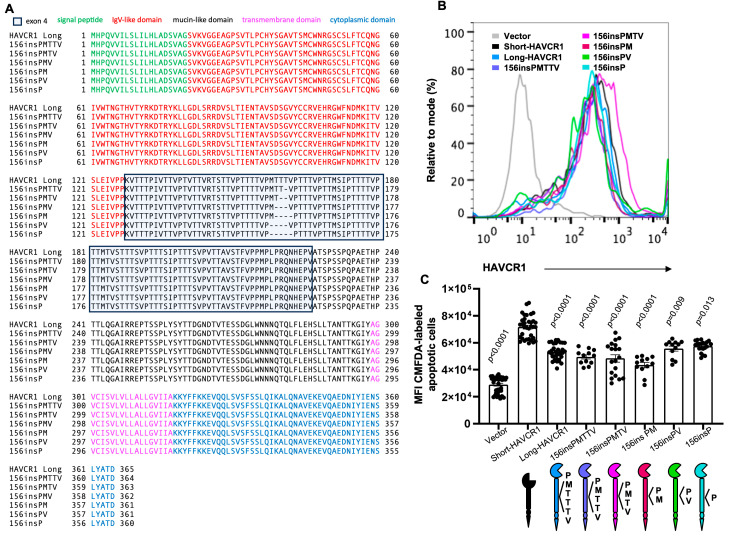
Effect of the peptide length insertion at position 156 of HAVCR1 in modulating binding of apoptotic cells to the IgV domain. (**A**) Amino acid sequence alignment as in Figure 1B of the long-HAVCR1 (156insPMTTTV) natural variant and HAVCR1 constructs containing PMTTV, PMTV, PM, PV, or P insertions at position 156. (**B**) Flow cytometry analysis of the expression of HAVCR1 at the cell surface of 293 H cells stably transfected with cDNA of short-HAVCR1 (black line) or constructs from (**A**) containing insertions at position 156 (colored lines). (**C**) Quantitation of binding of apoptotic cells to 293 H stable transfectants expressing HAVCR1 constructs. Binding of CMFDA-labeled Jurkat apoptotic cells to 293 H stable transfectants shown in (**B**) as described in Figure 2C. Data are mean ± s.e.m. of at least four independent experiments with three biological replicates per each HAVCR1 construct. *p*-values between short-HAVCR1 and the other cell transfectants were determined by one-way ANOVA with Dunnett’s test analysis.

Our data clearly showed that the PMTTTV insertion at HAVCR1 position 156 reduced binding of apoptotic cells to the IgV. To determine the minimum size of the insertion required to reduce binding of apoptotic cells, we compared binding of apoptotic cells to HAVCR1 mutants containing insertions of 1 to 6 amino acid of the hexamer repeat at position 156 (Figure 3A). Stable 293 H cell transfectants expressing similar levels of the HAVCR1 insertion mutants 156insPMTTV, 156insPMTV, 156insPM, 156insPV, and 156insP at the cell surface (Figure 3B) bound equivalent amounts of apoptotic cells comparable to long-HAVCR1, which contains the complete PMTTTV insertion at position 156 (Figure 3C). All these insertion mutants bound significantly lower levels of apoptotic cells than short-HAVCR1. Therefore, the reduced binding of apoptotic cells to HAVCR1 was not affected by the length of the insertion, requiring only a P at position 156.

### 3.4. The Insertion of Multiple P Residues at Position 156 Increase Binding of Apoptitic Cells to the IgV Domain of HAVCR1

**Figure 4:**
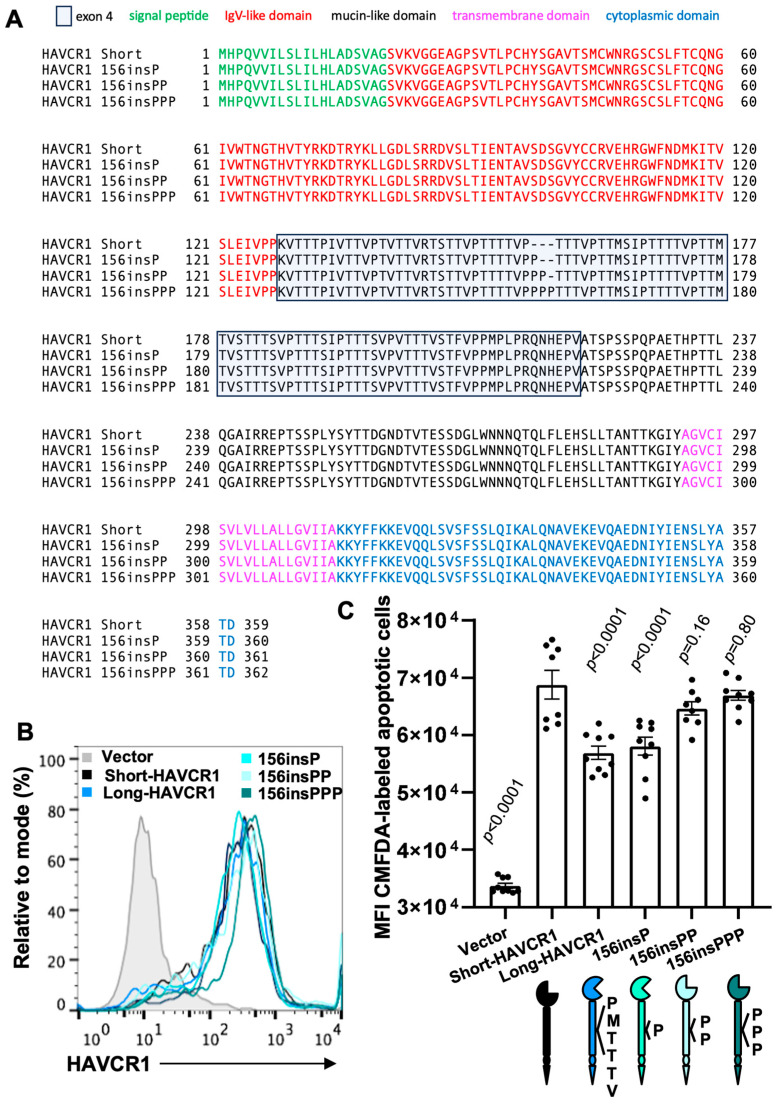
Insertion of two or three P residues at position 156 of short-HAVCR1 increased binding of apoptotic cells. (**A**) Amino acid sequence alignment as in Figure 1B of short-HAVCR1 and short-HAVCR1 constructs containing insertions at position 156 of P (156insP), PP (156insPP), or PPP (156insPPP). (**B**) Flow cytometry analysis of the expression of HAVCR1 on 293 H cells stably transfected with vector (filled grey histogram) or the cDNA of short-HAVCR1 (black line), long-HAVCR1 (blue line), or HAVCR1 156ins constructs containing one (green line), two (light-blue), or three (turquoise line) extra P residues. (**C**) Binding of apoptotic cells to 293 H cell transfectants from (**A**). Binding of CMFDA-labeled Jurkat apoptotic was performed as in Figure 2C. Data are mean ± s.e.m. of three independent experiments with three biological replicates each. *p*-values between short-HAVCR1 and the other cell transfectants were determined by one-way ANOVA with Dunnett’s test analysis.

All amino acids contain similar main carbon chains, which form peptidyl bonds with other amino acids, and diverse side chains or *R* groups of different lengths and specific characteristics. Because P is the only natural amino acid in which the R group forms a ring that includes the main chain N atom, it results in bending of the protein main chain [64]. To analyze the role of P residues in the HAVCR1 Muc, we constructed HAVCR1 mutants containing insertions of one (156insP), two (156insPP), or three (156insPPP) P residues at position 156 of the Muc (Figure 4A). Stable 293 H cell transfectant clones expressing similar levels of the HAVCR1 mutants at the cell surface (Figure 4B) were selected by flow cytometry. Cell transfectants expressing HAVCR1 156insPP and 156insPPP bound similar levels of apoptotic cells to short-HAVCR1 (Figure 4C). Cell transfectants expressing 156insP and long-HAVCR1 bound significantly lower levels of apoptotic cells. These binding data indicate that the protein bend induced by a single extra P residue at position 156 of the HAVCR1 Muc reduced binding of apoptotic cells to the IgV, whereas an extra two and three P residues at the same position, which are associated with protein loops and turns [65], induced further bending of the Muc reorienting the IgV domain into a more favorable conformation for binding apoptotic cells.

### 3.5. Apoptotic Cell Binding and Phocytosis Does Not Require Phosphorylation of the HAVCR1 Cytoplasmic Tail

The cytoplasmic tail of HAVCR1 contains two tyrosine (Y) residues (Figure 1A,B): Y356 lies within a conserved tyrosine kinase phosphorylation motif for SRC kinases, whereas Y362 has no apparent consensus phosphorylation motif [1,66]. Phosphorylation of the conserved Y residue and downstream signaling are induced by chemical activation and antibody crosslinking of HAVCR1 [2] and its mouse ortholog havcr1 [1,67]. Binding of apoptotic cells to HAVCR1 also triggers phosphorylation of the conserved Y residue and signaling through the phosphatidylinositol-3-kinase/protein kinase B (PI3K/AKT) pathway [10]. However, it is unknown whether phosphorylation of the HAVCR1 Y356 is required for binding and phagocytosis of apoptotic cells.

Upon binding to HAVCR1 at the cell surface, apoptotic cells are endocytosed and transported to an endosomal acidic compartment [7]. To investigate whether phosphorylation of HAVCR1 Y356 and Y362 are required to phagocytose apoptotic cells, we mutated each Y residue (Y356A or Y362A) or both (Y356/362A) to A in long-HAVCR1.

**Figure 5:**
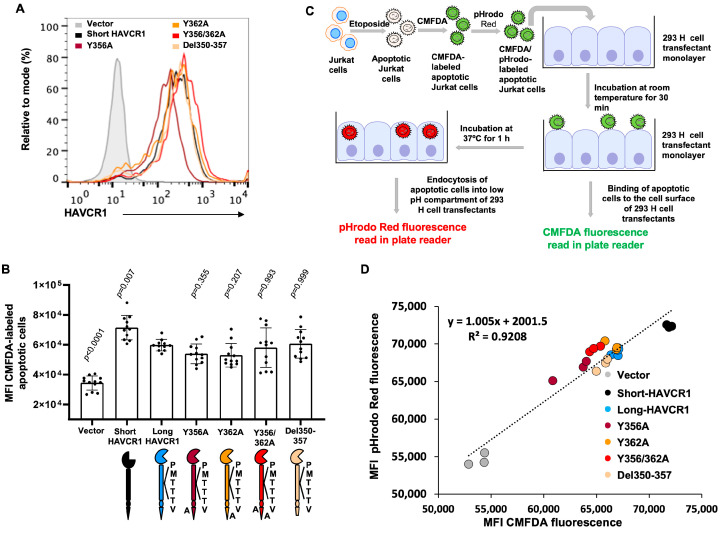
Phosphorylation of HAVCR1 cytoplasmic tail did not affect binding or internalization of apoptotic cells. (**A**) Flow cytometry analysis of the expression of HAVCR1 at the cell surface of 293 H cells stably transfected with vector (filled grey histogram) or the cDNA of short-HAVCR1 (black line), long-HAVCR1 (blue line), or HAVCR1 constructs containing a deletion of amino acids 350 to 357 (Del350-357, yellow line) or Y to A mutations at position 356 (Y356A, brown line), 362 (Y362A, orange line), or both 356 and 362 (Y356/362A, red line). (**B**) Binding of apoptotic cells to 293 H cell transfectants from (**A**). Binding of CMFDA-labeled apoptotic Jurkat cells was performed as in Figure 2C. Data are mean ± s.e.m, *n* = 12 from four independent experiments with three biological replicates each. *p*-values between long-HAVCR1 and the other cell transfectants were determined by one-way ANOVA with Dunnett’s test analysis. (**C**) Schematic representation of the combined binding and endocytosis assay. Apoptotic Jurkat cells labeled with CMFDA and pHrodo red were added to subconfluent monolayers of 293 H cell transfectants from (**A**), incubated for 30 min at RT, washed extensively, and incubated for 1 h at 37 °C. CMFDA (bound apoptotic cells) and pHrodo red (phagocytosed apoptotic cells) fluorescence were quantified in a microplate reader after 30 min incubation at RT and 1 h incubation at 37 °C, respectively. (**D**) Correlation between binding of apoptotic cells to HAVCR1 and endocytosis. Data from the combined binding and endocytosis assay described in (**C**) for each cell transfectant determined in triplicates (dots). Regression analysis was performed using the Excel program, and showed a linear relationship between binding end endocytosis of apoptotic cells. R^2^ is regression coefficient.

We also deleted the conserved tyrosine kinase phosphorylation site between amino acids 350 to 357 (Del350-357). Clones of 293 H cell transfectants that expressed similar levels of the HAVCR1 mutants and long-HAVCR1 at the cell surface (Figure 5A) bound equivalent amounts of apoptotic cells (Figure 5B). As expected, short-HAVCR1 bound significantly higher amounts of apoptotic cells, whereas vector transfected cells only bound background levels. These binding data confirmed that phosphorylation of the cytoplasmic tail of HAVCR1 is not required for the interaction of PS with the IgV binding domain. 

To investigate the effect of the HAVCR1 cytoplasmic tail mutations in phagocytosis of apoptotic cells, we performed a combined binding and endocytosis assay (Figure 5C) using apoptotic Jurkat cells double-stained with pHrodo red, a dye that requires internalization into an acidic compartment to fluoresce and is commonly used to track phagocytosis, and CMFDA, which is pH-insensitive and fluoresces at the cell surface and within cells. In this combined binding and endocytosis assay, binding of apoptotic cells to the cell surface of the 293 H cell transfectants was determined in a fluorescence plate reader after 30 min incubation at RT using CMFDA-specific filters, then the temperature was shifted to 37 °C for 1 h, and endocytosis of apoptotic cells was determined using pHrodo Red-specific filters. Figure 5D shows a linear relationship between bound (CMFDA fluorescence) and endocytosed (pHrodo Red fluorescence) apoptotic cells. Cell transfectants expressing short-HAVCR1 bound and phagocytosed the highest levels of apoptotic cells compared to cells expressing long-HAVCR1 and cytoplasmic tail mutants, whereas vector transfectants only bound and phagocytosed background levels of apoptotic cells. Taken together, this combined binding and endocytosis assay showed that mutations in the cytoplasmic tail of HAVCR1 did not affect HAVCR1-mediated phagocytosis of apoptotic cells and that tyrosine-phosphorylation is not required for phagocytosis of apoptotic cells bound to the HAVCR1 IgV.

### 3.6. Short-HAVCR1 Induces Stronger PI3K/AKT/mTOR Cell Signaling Than Long-HAVCR1 

To analyze HAVCR1-mediated cell signaling, we used a minimalistic approach based on the activation of HAVCR1 with liposomes containing PS, which binds to the HAVCR1 IgV MILBS, compared to control liposomes that only contain PC and do not bind to HAVCR1 [7]. Treatment of 293 H cell transfectants expressing HAVCR1 with PC:PS liposomes induced cell signaling that resulted in the phosphorylation of PI3K and AKT, whereas treatment with PC liposomes had no effect (Figure S1A,B). Similarly, treatment with PC:PS liposomes induced the phosphorylation of PI3K and AKT in Jurkat cells expressing HAVCR1 (Figure S2), whereas PC liposomes failed do so (Figure S1C,D). These activation data indicated that HAVCR1-mediated cell signaling required the presence of PS in the liposomes but was independent on the cell line used in the experiment. As expected, PC:PS or PC liposomes did not induce phosphorylation of PI3K or AKT in vector-transfected 293 H and Jurkat cells (Figure S1A–D), indicating that cell signaling induced by the PC:PS liposomes depended on HAVCR1 expression. 

**Figure 6:**
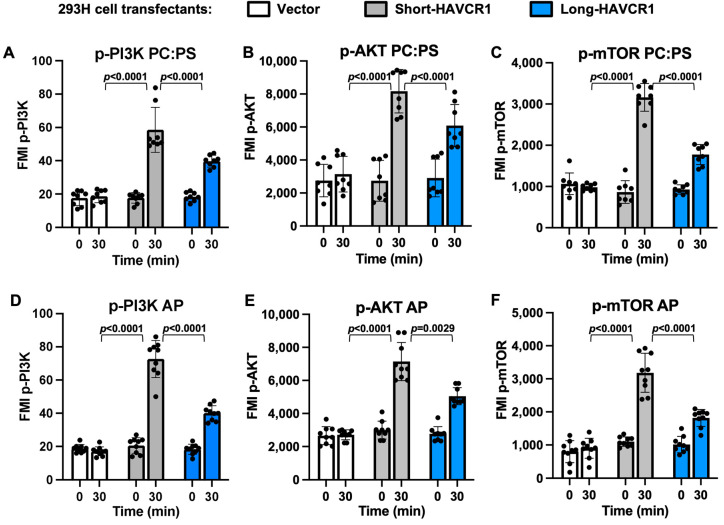
Cell signaling of 293 H cell transfectants activated with liposomes or apoptotic cells. (**A**–**C**) Activation of 293 H cell transfectants with liposomes containing PS. Cell extracts of 293 H cells stably transfected with vector (white bars) or cDNA of short-HAVCR1 (grey bars) or long-HAVCR1 (blue bars) from Figure 1C and treated with PC:PS liposomes for 0 or 30 min were analyzed using a multiplex cell signaling assay to quantitate phosphorylation of specific proteins. (**D**–**F**) Cell extracts of 293 H cell transfectants as in (**A**–**C**) were treated with apoptotic Jurkat cells (AP) for 0 or 30 min and analyzed using the same multiplex signaling assay as in (**A**–**C**). Data are fluorescence mean intensity (MFI) ± s.d., *n* = 8 or 9 from three independent experiments with two to three biological replicates each of phosphorylated PI3Kp85 at tyrosine 458 (p-PI3K) (**A**,**D**), AKT at serine 473 (p-AKT) (**B**,**E**), and mTOR at serine 2448 (p-mTOR) (**C**,**F**). *p*-values between short-HAVCR1 and the other cell transfectants were determined by two-way ANOVA with Tukey’s test analysis.

**Figure 7:**
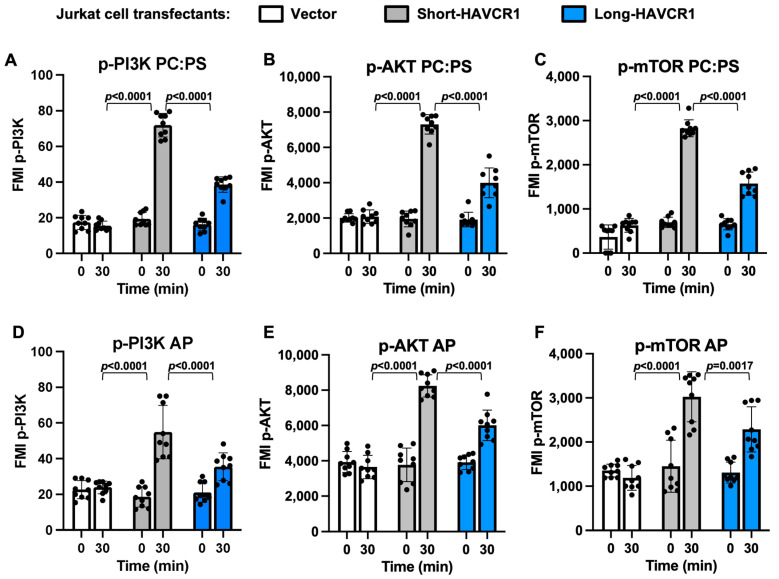
Cell signaling of Jurkat cell transfectants activated with liposomes or apoptotic cells. (**A**–**C**) Activation of Jurkat cell transfectants with liposomes containing PS. Cell extracts of Jurkat stably transfected with vector (white bars) or cDNA of short-HAVCR1(grey bars) or long-HAVCR1 (blue bars) from Figure S2 and treated with PC:PS liposomes for 0 or 30 min were analyzed using a multiplex cell signaling assay to quantitate phosphorylation of specific proteins. (**D**–**F**) Cell extracts of Jurkat cell transfectants as in (**A**–**C**) were treated with apoptotic Jurkat cells (AP) for 0 or 30 min and analyzed using the same multiplex signaling assay as in (**A**–**C**). Data are fluorescence mean intensity (MFI) ± s.d., *n* = 9 from three independent experiments with three biological replicates each of phosphorylated PI3Kp85 at tyrosine 458 (p-PI3K) (**A**,**D**), AKT at serine 473 (p-AKT) (**B**,**E**), and mTOR at serine 2448 (p-mTOR) (**C**,**F**). *p*-values between short-HAVCR1 and the other cell transfectants were determined by two-way ANOVA with Tukey’s test analysis.

Because 293 H cells expressing short-HAVCR1 bound and endocytosed higher levels of apoptotic cells compared to cells expressing long-HAVCR1, we hypothesized that binding of PS to the IgV of the former would also induce a stronger cell signaling than to the latter. To test our hypothesis, we analyzed cell signaling in 293 H cell transfectants expressing short-HAVCR1, long-HAVCR1, or vector that were activated for 0 or 30 min with PC:PS liposomes (Figure 6A–C). A multiplex assay that analyzed phosphorylation of PI3K, AKT, and mTOR, which is phosphorylated by activated AKT, showed that the PC:PS liposomes induced stronger cell signaling in cells expressing short-HAVCR1 than long-HAVCR1, whereas no signaling was detected in vector transfectants. To analyze cell signaling in a more biologically relevant system, we activated transfectants with apoptotic cells (Figure 6D–F), which showed similar results than the activation with PC:PS liposomes. A similar pattern of cell signaling was observed in Jurkat cells expressing similar levels of short- and long-HAVCR1 at the cell surface (Figure S2) activated with PC:PS liposomes (Figure 7A–C) or apoptotic cells (Figure 7D–F). These activation data indicated that short-HAVCR1 induced stronger cell signaling in cells of immune (Jurkat cells) and non-immune (293 H cells) lineages. Taken together, our results indicate that the dampening effect of the 156insPMTTTV in the binding of apoptotic cells also affected HAVCR1-mediated cell activation.

## 4. Discussion

Our work focused on understanding the molecular basis for the differential function of human *HAVCR1* exon 4 ins/del polymorphisms, which have been associated with immune and infectious diseases. Because HAVCR1 is expressed in both immune and non-immune cells, the association of HAVCR1 exon 4 ins/del with disease is multifaceted and complex. 

Soon after we discovered monkey and human HAVCR1 in the 1990s [4,5], it became apparent that this receptor was highly polymorphic [33]. The high degree of polymorphism and unusual divergence between human, chimp, and gorilla sequence in *HAVCR1* exon 4 suggest that evolutionary selective pressures acted on the Muc region of this gene [34]. Several *HAVCR1* exon 4 ins/del polymorphisms have been identified that affect the length of the Muc domain. The first human HAVCR1 variant identified contained 13 hexamer repeats [5] and is referred in this work as short-HAVCR1. A HAVCR1 variant containing five amino acid insertions at position 156 has also been described [34] and is referred in this paper as 156insPMTTV or intermediate-HAVCR1. The longest form of HAVCR1 contains an insertion of an extra PMTTTV hexamer repeat at position 156 and is referred to in this work as long-HAVCR1 or 156insPMTTTV [60]. This long-HAVCR1 has also been reported in the literature as 157insMTTTVP, but we believe that the 156insPMTTTV nomenclature is more accurate because of historical data showing that the Muc repeats start at a proline residue [4,5]. Here, we showed that the addition at position 156 of a single P residue is sufficient to reduce binding of apoptotic cells to HAVCR1 and the magnitude of HAVCR1-mediated cell signaling, which provides further evidence that a P residue could be considered as the first amino acid of each repeat.

The association of HAVCR1 polymorphisms with infectious, allergic, and autoimmune diseases (for reviews, see [15,68]) provided further evidence that evolutionary pressure impacted the natural selection of HAVCR1 variants. However, these associations are not universal and depended on the studied populations [68] suggesting that differences in genetic backgrounds and environmental interactions also modulate HAVCR1 function. 

Polymorphisms in *HAVCR1* exon 4 have been associated with susceptibility to infectious diseases. Long-HAVCR1, which binds HAV more efficiently than short-HAVCR1, was associated with increased severity of hepatitis A in Argentinean [31] but not in Indian children [58]. CD4+ T cells that express short-HAVCR1 have a lower rate of HIV-1 infection in vitro that was associated with protection from HIV-1 infection in exposed seronegative individuals [57]. Taken together, the current data on the association between susceptibility to infection and polymorphisms in HAVCR1 exon 4 suggest that short-HAVCR1 plays a protective role and explain the high evolutionary pressure on *HAVCR1* exon 4 towards shorter variants [34]. 

Pioneer work by McIntire et al. [60] showed an association between long-HAVCR1 and protection against atopy in HAV seropositive individuals in the US. These authors hypothesized that the HAV infection permanently affected Th2 responses, preventing its dysregulation and the onset of atopy. A study based on the Japanese population, in which short-HAVCR1 is predominant (86% of the population) and the incidence of HAV infection is very low, showed no association between protection against atopy and long-HAVCR1, further suggesting that HAV infection of an individual carrying long-HAVCR1 can protect against atopy [69]. In African Americans, who predominantly carry the long-HAVCR1 allele (64% of the population), short-HAVCR1 was associated with protection against asthma independently of the HAV infection status [61]. Intermediate-HAVCR1 containing the five amino acids insertion 156insPMTTV has also been associated with atopy in US children with unknown HAV infection status [70]. Taken together, these data suggest that short-HAVCR1 is protective, whereas long-HAVCR1 induces atopy in humans. 

The role of the HAVCR1 156ins/delPMTTTV polymorphisms in autoimmunity has been more difficult to assess due to the nonrandom association with other polymorphisms in *HAVCR1* [46,49]. Consequently, the separate contribution of individual ins/del could not be assessed with confidence due to their linkage disequilibrium [49]. The analysis of haplotypes instead of single polymorphisms revealed that patients with RA have higher mRNA expression levels of short-HAVCR1 but different SNPs in *HAVCR1* introns 4, 5, 6, 7, and 8. These haplotype data in RA patients indicate that the mRNA level, in addition to ins/del in exon 4, is responsible for the association of HAVCR1 with RA [49], and suggest that long-HAVCR1 is protective against autoimmune diseases. Because of the association short-HAVCR1 with RA, a Th1- and Th17-mediated disease, and long-HAVCR1 with atopy, a Th2-mediated disease, it is likely that the *HAVCR1* 156ins/del in exon 4 are capable of polarizing immune responses.

Although the association between *HAVCR1* exon 4 variants with immune and infectious diseases has been well established, the molecular basis for the differential function of the HAVCR1 variants has not been explored. In this work, we showed that short-HAVCR1 binds more apoptotic cells than long-HAVCR1 (Figure 1), which indicated that insertions in the Muc domain affect how the IgV binding domain is presented at the cell surface. Because membrane-bound receptors can interact in *trans* with cell-surface ligands on other cells and in *cis* with ligands from the same cells [71], it is possible that the 156 ins/del in HAVCR1 exon 4 affected the *cis/trans* interactions of HAVCR1. While short-HAVCR1 may favor *trans* interactions of the IgV with apoptotic cells, long-HAVCR1 may limit the availability of the IgV binding domain for *trans* interactions. A plausible explanation is that the insertion of an extra repeat at position 156 of the Muc domain bended the extracellular domain of HAVCR1 towards the plasma membrane favoring ***cis*** interaction of the IgV domain with cell membrane phospholipids. However, it is also possible that insertions at position 156 allowed the IgV and/or Muc to interact with other cell surface molecules that affected the HAVCR1 phenotype. Further research will be required to determine whether the insertions at position 156 favor the interaction of HAVCR1 with phospholipids, proteins, or other molecules at the cell surface.

Activation of HAVCR1 triggers the PI3K/AKT pathway [1,2], which is a conserved signaling network interconnected with other signaling pathways that modulates cell survival, growth, and differentiation (for a review, see [3]). In this study, we looked at the PI3K/AKT/mTOR pathway but not at other immune-related signaling pathways such as NF-κB or MAPK, which should be the subject of future research. Upon activation, a conserved Y residue in the cytoplasmic tail of HAVCR1 is phosphorylated by Src-family kinases [1] resulting in the recruitment and phosphorylation of PI3K [2]. Activation of PI3K converts phosphatidylinositol (3,4)-bis-phosphate (PIP2) lipids into phosphatidylinositol (3,4,5)-tris-phosphate (PIP3), which is present at the inner leaflet of the plasma membrane. Binding of AKT to PIP3 induces AKT phosphorylation and the indirect activation and phosphorylation of mTOR triggering cell growth and division (for reviews, see [72,73]). We have shown that binding of apoptotic cells to HAVCR1 activates AKT and prevents cell death [10]. Here we expanded our studies to analyze the role of *HAVCR1* exon 4 polymorphisms in the activation of the PI3K/AKT/mTOR pathway and found that short-HAVCR1 induced stronger PI3K/AKT/mTOR cell signaling than long-HAVCR1 (Figure 6 and Figure 7). We also found that there is a direct correlation between binding of apoptotic cells and the magnitude of the PI3K/AKT/mTOR signaling, i.e., short-HAVCR1 bound more apoptotic cells and induced stronger cell signaling than long-HAVCR1 (Figure 5D). Because of the association of short-HAVCR1 with RA, our data suggest that the strong ligand binding and signaling mediated by short-HAVCR1 could drive polarization of immune cells towards Th1/Th17 responses enhancing pro-inflammatory responses. Additional studies will be required to determine whether immune polarization is mediated by the magnitude of HAVCR1-related cell signaling.

Our work did not provide a plausible explanation that justified the evolutionary preference for maintaining the ins/del of multiple residues at position 156 of HAVCR1. To the contrary, we showed that shifting the insertion one repeat towards the N-terminus (150insPMTTTV) or the C-terminus (161insPMTTTV) of HAVCR1 also reduced binding of apoptotic cells to HAVCR1 (Figure 2). This shifting experiment suggests there is no stringent positional requirement to maintain the reduced binding phenotype of long-HAVCR1. Furthermore, we showed that the additions of a single extra P residue inserted at position 156 was sufficient to reduce binding of apoptotic cells to HAVCR1 (Figure 3 and Figure 4), indicating that additional residues are not required to reduced binding of apoptotic cells to long-HAVCR1. Consequently, we concluded that unknown determinants drive the evolutionary pressure to maintain the ins/del of multiple residues at position 156 of HAVCR1. 

In summary, our work focused on the effect of *HAVCR1* exon 4 polymorphisms in binding of apoptotic cells to the IgV domain of HAVCR1 and cell signaling. Our studies found that the insertion of an extra hexamer repeat at position 156 of the HAVCR1 Muc reduced binding of apoptotic cells to the IgV and the magnitude of the HAVCR1-mediated signaling. These binding and cell signaling results indicate that the 156ins/del modulate how the IgV-binding domain of HAVCR1 is presented at the cell surface and suggest that short- and long-HAVCR1 can skew immune responses towards Th1/Th17 and Th2 polarity, respectively. Further work will be needed to substantiate our findings in vivo, which could support targeting HAVCR1 variants to modulate atopic and autoimmune responses using antibodies, small molecules, and gene therapy approaches.

## Data Availability

The raw data supporting the conclusions of this article will be made available by the authors on request.

## Supplementary Materials

The following supporting information can be downloaded at: https://www.mdpi.com/article/10.3390/biomedicines12112643/s1, Figure S1: HAVCR1-mediated cell signaling in 293 H and Jurkat cell transfectants activated with liposomes; Figure S2: Expression of long- and short-HAVCR1 at the cell surface of Jurkat cell transfectants.
