# Supplementary material for: Molecular Basis for the Differential Function of HAVCR1 Mucin Variants
Source: Biomedicines. 2024 Nov 19;12(11):2643. doi: 10.3390/biomedicines12112643 (PMC11592376; doi:10.3390/biomedicines12112643)
Supplement: Supplementary file 1 [file biomedicines-12-02643-s001.zip › biomedicines-3268192-supplementary.pdf]

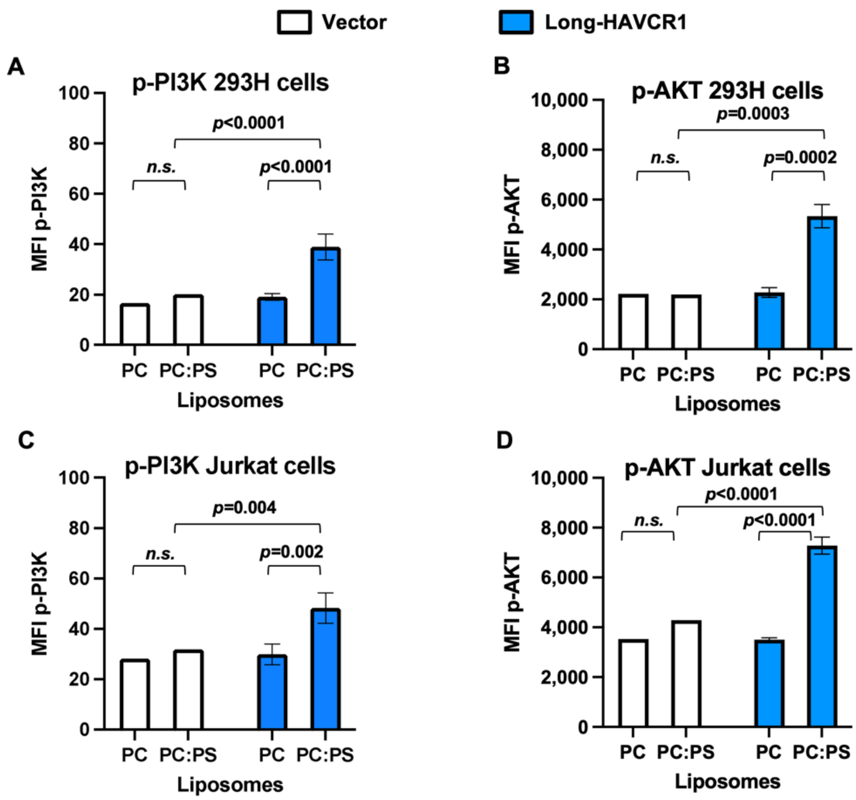

Figure S1. HAVCR1-mediated cell signaling in 293 H and Jurkat cell transfectants activated with liposomes. Cell signaling of 293 H (**A, B**) and Jurkat (**C, D**) cells stably transfected with vector (white bars) or the cDNA of long-HAVCR1 cDNA (blue bars) and activated with liposomes containing PC or PC:PS for 0 or 30 min. Cell extracts of activated cells were analyzed using a multiplex cell signaling assay to quantitate phosphorylation of PI3Kp85 at tyrosine 458 (p-PI3K) (**A,C**) and AKT at serine 473 (p-AKT) (**B,D**). Data are fluorescence mean intensity (MFI) ± s.d., *n*=3, from a representative experiment. P values between cell transfectants were determined by two-way ANOVA with Tukey's post-test analysis. Nonsignificant MFI differences (*n.s.*).

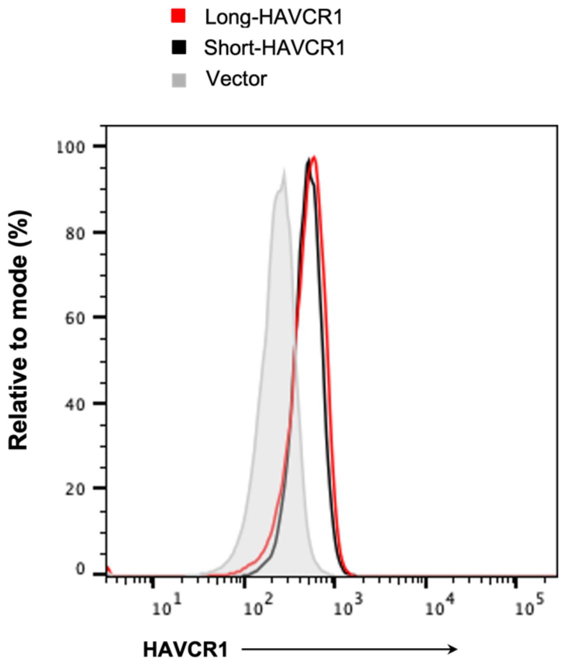

Figure S2. Expression of long- and short-HAVCR1 at the cell surface of Jurkat cell transfectants. Flow cytometry analysis of the expression of HAVCR1 at the cell surface of Jurkat cells stably transfected with vector (filled grey histogram) or the cDNA of short-HAVCR1 (black line) or long-HAVCR1 (blue line) stained with anti-HAVCR1 mAb 1D12.
